# Fumaric Acid and its Esters: An Emerging Treatment for Multiple Sclerosis

**DOI:** 10.2174/157015909787602788

**Published:** 2009-03

**Authors:** D Moharregh-Khiabani, R.A Linker, R Gold, M Stangel

**Affiliations:** 1 Department of Neurology, Medical School Hannover, Carl-Neuberg-Strasse 1, D-30625 Hannover, Germany;; 2 Department of Neurology, St. Josef-Hospital/Ruhr-University Bochum, Gudrunstrasse 56, D-44791 Bochum, Germany

**Keywords:** Fumaric acid, immunomodulation, multiple sclerosis, neuroprotective effects, phase II study.

## Abstract

Fumaric acid is an intermediate product of the citric acid cycle that is a source of intracellular energy in the form of adenosine triphosphate (ATP). It is generated by oxidation of adenylsuccinate by the enzyme succinate dehydrogenase and is then converted to maleate by the enzyme fumarase. At present, fumaric acid esters (FAE) are licensed for the treatment of psoriasis. Several lines of evidence have demonstrated immunomodulatory effects for FAE. Clinical studies in psoriasis showed a reduction of peripheral CD4^+^- and CD8^+^-T-lymphocytes due to the ability of FAE to induce apoptosis. *In vitro *studies with the ester dimethyl fumarate (DMF) described an inhibitory effect on nuclear factor kappa B (NF-κB)-dependent transcription of tumor necrosis factor-alpha (TNF-α) induced genes in human endothelial cells. Animal studies using a model of central nervous system demyelination, MOG-induced experimental autoimmune encephalomyelitis (EAE), revealed a reduction of microglia and macrophages in inflamed lesions. A phase II clinical study in relapsing-remitting multiple sclerosis (RRMS) patients with a modified fumaric acid ester, BG-12, showed as "proof of principle" a significant reduction in the number of gadolinium enhancing lesions after 24 weeks of treatment as compared to placebo. Further phase III studies have now started to explore the long-term efficacy of FAE.

## INTRODUTION

Multiple sclerosis (MS) is the most common cause of neurological disability in young adults. The current treatment options for relapsing-remitting MS (RRMS) are only partially effective and require a parenteral route of administration. Therefore, there is a need for new and well tolerated, preferably orally available therapeutics.

Fumaric acid esters (FAE) are a group of simple structured compounds that have been used in the treatment of psoriasis since 1959, originally proposed by the german chemist Schweckendiek [23]. He himself suffered from this disease and assumed that a disturbance in the citrate cycle might be the cause, since immune cells are so critically dependent on energy supply. Over the next decades, a mixture of compounds consisting of dimethylfumarate (DMF) and three salts of ethylhydrogenfumarate (EHF) was developed to reduce (mainly gastrointestinal) side effects and was licensed in Germany in 1994 as oral therapy for serve psoriasis under the brand name Fumaderm^®^. Over the past 15 years, there have been many clinical trials that demonstrated the immunomodulatory efficacy and safety of oral FAE in this indication [3, 9, 15]. Due to its immunomodulatory potential, FAE are also evaluated as a potential treatment for RRMS. Here we summarize the rationale for such investigations, the known mechanisms of action, and the results of early clinical trials.

## TREATMENT OF FAE IN PSORIASIS

Fumaderm^®^, an enteric-coated tablet, has become the most prescribed systemic treatment for psoriasis in Germany since 1994 [20]. A total of 13 studies confirmed that a proportion of 50-70% of the patients showed an improvement of approximately 75% of the baseline “psoriasis area and servitiy index” (PASI) after 4 month of treatment [13, 15, 16, 17]. Although there are some side effects like flushing, nausea, vomiting and diarrhea in 30% of patients which typically limit the treatment period to 4-6 weeks, Fumaderm^®^ showed an excellent antipsoriatic effect in clinical studies and appears to be fairly safe in comparison with other systemic anti-psoriatic treatment options. Neither long-term toxicity, nor a higher risk for infections or malignancies have been observed [7]. Furthermore, there are no described interactions with other drugs, except those known to have damaging effects on the kidneys.

## PHARMACOLOGICAL PROPERTIES OF FAE

After oral intake, DMF, the main component of Fumaderm^®^, is rapidly hydrolysed by esterases to its metabolite monomethyl fumarate (MMF). After complete absorption in the small intestine [29], it can interact with immune cells in the blood circulation [10]. MMF, the most bioactive metabolite [18], is further metabolized in the citrate cycle to carbon dioxide and water and finally eliminated mainly though breathing while only small amounts of intact MMF are excreted through urine or faeces. The half-life of DMF is about 12 minutes and 36 hours for MMF. The highest concentration of MMF in human serum is measured 5 to 6 hours after oral intake. There is no evidence for a cytochrome P450 dependent metabolism in the liver [16].

## MODE OF ACTION

Despite numerous *in vitro* and *ex vivo* studies, the mechanism of action of FAE is not fully understood. A convenient hypothesis is based on the idea that DMF interferes with the cellular redox system by modulating intracellular thiols and thereby increasing the level of reduced glutathione [14]. These increased glutathione levels may finally lead to an inhibition of the translocation of NF-κB into the nucleus. Altering the NF-κB pathway results in a decreased expression of NF-κB dependent genes that regulate the expression of a cascade of inflammatory cytokines, chemokines, and adhesion molecules [25]. This affects different types of cells in the immune system (Table **1**) and their counterparts like the endothelium. However, at higher concentrations, DMF may induce apoptosis in all cell types.

### Effect of FAE on T-Cells

One of the first observations during the application of Fumaderm^®^ in psoriasis was the effect on T-cells. Initial studies revealed a decrease of T-cells in nearly all treated patients [2]. This finding was well in line with an immunohistochemical study that showed a reduction of CD4^+^-cells by half [5] in the epidermal inflammatory infiltrate. Subsequent *in vitro* studies confirmed the capability of DMF to induce apoptosis in human T-cells [26]. Upon analysis of the cytokine production in more detail, de Jong* et al.* [6] reported that MMF increased the production of the “TH2” cytokines interleukin (IL)-4 and IL-5 in stimulated T-cells without having an effect on the “TH1” cytokine interferon gamma (IFN-γ), the IL-2 production, or the proliferation of T-cells. In another study, stimulated CD4^+^CD45RO^+^ memory T-cells again showed an increased secretion of IL-4 and IL-5 after treatment with MMF. Taken together, these findings suggest that FAE shift the cytokine profile from a “TH1” to a “TH2” profile. This effect of FAE on the expression of cytokines may also apply to other cell types than T-cells.

### Effects of FAE and Peripheral Blood Mononuclear Cells (PBMC)

In human PMBC stimulated either with IFN-γ or lipopolysaccharide (LPS) the expression of the chemokines CXCL8, CXCL9, and CXCL10 was dose dependently inhibited by DMF [19]. Application of MMF led also to a higher expression of IL-4, IL-5, TNF-α, IL-10 and IL-1RA which is well in line with the findings in T-cells [4]. In monocytes, FAE induced an upregulation of the production of superoxide anions [32].

### Effects of FAE on B-Cells

Although there have not been any studies on direct FAE effects on B-cells, there is evidence that the downregulation of NF-κB can inhibit the anti-apoptotic protein Bcl-2 which can in turn lead to apoptosis in B-cells [14]. It is thus tempting to speculate that FAE mediated inhibition of NF-κB activity may also affect B-cell functions and influence B-cell apoptosis.

### Effects of FAE in Keratinocytes

Keratinocytes are themselves expressing cytokines and chemokines and are part of the inflammatory response in psoriasis. Thus, their response to FAE was of great interest. Ockenfels* et. al.* [19] demonstrated that in keratinocytes co-cultured with a T-cell line, DMF can increase the expression of IL-10 and inhibit IFN-γ, IL-6 and transforming growth factor (TGF)-α. Another study with the keratinocyte cell line HaCat showed that DMF suppresses the expression of intercellular adhesion molecule-1 (ICAM-1) and HLA-DR [24]. Recent studies revealed that DMF can also inhibit the expression of the chemokines CXCL1, CXCL8, CXCL9, CXCL10 and CXCL11 in keratinocytes [25] which are to different degrees involved in chemoattraction of neutrophilic granulocytes, macrophages and T-cells.

### FAE and Dendritic Cells (DC)

Dendritic cells play a major role in regulating inflammatory responses in autoimmune diseases like psoriasis and MS by expressing cytokines and co-stimulatory molecules. In monocyte derived DC, FAE prevented appropriate cell differentiation [31]. In another study, MMF treated DC altered the lymphocyte response to a “TH1” profile after stimulation by lipopolysaccharide (LPS) [11].

### FAE Effects on Endothelial Cells

In experiments with human umbilical vein endothelial cell cultures, Asadullah* et al. *and Vandermeeren* et al.* showed that DMF inhibits the tumor necrosis factor (TNF)-α induced expression of ICAM-1, E-selectin, and the vascular cell adhesions molecule-1 (VCAM-1) [4, 27].

### Effects of FAE on Glial Cells

A potential detoxification effect was found when DMF was tested in astrocytes and microglia [30]. DMF decreased the LPS induced production of proinflammatory cytokines like TNF-α, IL-1β, IL-6, and nitric oxide. Moreover, DMF increased the expression of the NAD(P)H: quinine reductase (NQO-1) and the content of cellular glutathione. Both are involved in glial detoxification pathways which could potentially add to a so far only poorly described neuroprotective effect of FAE.

## FAE IN EXPERIMENTAL AUTOIMMUNE ENCEPHALOMYELITIS (EAE)

Given the immunomodulatory properties of FAE, it was suggested that there may be also a beneficial effect in MS. Thus, Schilling *et al*. [21] investigated the effects of FAE in myelin oligodendrocyte glycoprotein (MOG) induced experimental autoimmune encephalomyelitis (MOG-EAE), an animal model mimicking several aspects of MS. Different dosages of FAE were administered by oral gavage starting from day 3 post immunization (p.i.). This study confirmed the positive effect of FAE. Histological analysis showed a significant reduction of microglia and macrophages, but not of T-cells at an early time point of the disease (day 26 p.i.) while cytokine analysis could not confirm a “TH1”-“Th2” shift. At later stages of the disease a significant preservation of myelin and axonal integrity was found. These effects correlated with the ability of FAE to induce Nrf2 dependent pathways which may mediate neuroprotective effects by increasing expression of NQO-1 and thus modulating the redox state in the inflamed central nervous system (CNS) [12].

## FAE IN MULTIPLE SCLEROSIS

Schimrigk *et al.* [22] completed a first exploratory, prospective, open-label study of FAE in 10 patients with RRMS. The investigations included safety and tolerability, the amount and volume of gadolinium-enhancing (Gd+) lesions on MRI, the relapse rate, changes in the Expanded Disability Status Scale (EDSS), the ambulatory index (AI), the nine-hole peg test (9-HPT), and a detailed cytokine profile. Inclusion parameters were the presence of at least one active lesion on baseline MRI, at least one relapse in the year before the beginning of the study, and an EDSS score between 2.0-6.0. There were four phases of the study: first a 6-week baseline monitoring phase, second an 18-week treatment phase, third a 4-week washout phase, and finally a second 48-week treatment phase. The patients were given a titrated maximum of 720 mg/d Fumaderm^®^ in the first phase and 360 mg/d in the second phase. FAE treatment led to a significant reduction of Gd+ lesions already after the first treatment phase. This effect persisted during the second treatment phase even with only half of the dose. The EDSS, AI and the 9-HPT remained stable or showed a slight improvement as compared to baseline. Immunological investigations revealed a small increase in the expression of IL-10 and a higher apoptosis rate of CD4^+^ lymphocytes. The main adverse events were gastrointestinal symptoms and flushing, all of which were reported as mild and reversible. These results supported the design of further clinical investigations.

In 2008 Kappos *et al.* [8] reported the results of a larger phase II study. This multicenter, double-blind, placebo-controlled study included 257 patients in 10 countries and was designed to evaluate the safety, efficacy, and dose-ranging. The study used a second generation fumaric acid derivate (BG-12) that was developed by Fumapharm AG and BiogenIdec Inc. and contains DMF [1, 28] as enteric-coated microtablet to improve gastrointestinal tolerability. The 257 patients were randomized in four groups to receive either placebo or BG-12 at 120, 360, 720 mg/d for 6 month, followed by another 6 month of a dose-blinded safety extension study where the placebo group received the highest dose of 720 mg/d. In this study, outcome parameters included the number and volume of Gd+ enhancing lesions, new or enlarging T2, new T1 lesions, and annualized relapse rate (Table **2**). In comparison to the placebo group, the high-dose BG-12 group displayed a statistically significant reduction of 69% in the mean number of Gd+ enhancing lesions as measured by monthly MRI between weeks 12 and 24. The 720 mg/d group also showed a reduction in newly enlarging T2 lesions of 48% and a reduction of 53% of T1 newly enlarging lesions as compared to the placebo group at week 24. Although the study was not designed to demonstrate an effect on the relapse rate, there was a trend toward a reduced annualized relapse rate by 32%. In the 120 mg/d and 360 mg/d treated groups the results were not statistically significant.

Adverse events included headache, mild infections, gastrointestinal symptoms, mild increase in liver enzymes and flushing which decreased during the second study phase and were all mild and reversible. In general, BG-12 appears to have a promising short-term efficacy and safety profile.

To determine the long-term safety and efficacy of BG-12, a phase III study program already started in Europe and North America. The program includes the DEFINE (determination of the efficacy and safety of oral fumarate in relapsing-remitting MS) and CONFIRM (comparator and an oral fumarate in relapsing-remitting MS) studies. These trials are designed as international, multicenter, two-year randomized, double-blind, placebo-controlled, dose comparison studies. The CONFIRM study will also contain a glatimer acetate group to compare BG12 with an established immunomodulatory therapy. These phase III studies will further elucidate the potential of BG12 in the treatment of RRMS.

## CONCLUSION

After the long experience in the treatment of psoriasis with more than 30000 patient years and several *in vitro* and animal studies, FAE seem a promising orally available option for the immunomodulatory treatment of patients with RRMS. Results from a phase II study support this potential. In particular, the new formulation BG12 displays an improved tolerability and an excellent safety profile. Experimental data suggest an immunomodulatory and an additional neuroprotective mechanism, making BG12 a promising target. The assumed immunomodulatory capacity will probably place BG12 in a similar range as the available injectable disease modifying drugs for treatment of RRMS in the early phase of the disease. Due to the excellent safety profile known from the use in psoriasis, BG12 may also prove to be a good candidate for combination therapies. The ongoing phase III clinical trials are designed to demonstrate the long-term efficacy in a large cohort of patients with RRMS. The results are eagerly awaited by both patients and physicians to satisfy the great need for new safe and orally available treatment options.

## CONFLICT OF INTEREST

This article was written without financial support. MS, RL, and RG have received honoraria for consulting services and lectures from Baxter, BiogenIdec, Biotest, Bayer, MerckSerono, Novartis, Sanofi-Aventis, Talecris, and Teva. MS and RG have received research support from BiogenIdec, Bayer, MerckSerono, Sanofi-Aventis, and Teva.

## Figures and Tables

**Table 1:** Summary of the Effect of FAE in Different Cell Types

Cell Type	Cytokine/Signaling Effect	MMF/DMF	Effect	References
T-cells	IL-10↑, IL-5↑,	MMF/DMF	“TH1” “TH2” shift, HO-1↑ reduced CD4^+^, CD8^+^numbers	[2, 5, 6, 26]
PBMC	CXCL8, 9, 10↓ TNF-α ↑, IL-10↑ IL-1RA↑ IL-4↑, IL-5↑	DMF	Superoxide anions↑	[4, 19, 32]
B-cells	NF- κB↓	n.d	Bcl-2↓, induce apoptosis	[14]
Keratinocytes	IFNγ↓,IL-10↓, IL-6↓, TGF-α↓, CXCL8, 9, 10, 11↓	DMF	HLA-DR↓,ICAM-1↓	[19, 24, 25]
Dendritic cells	Il-12↓,	MMF/DMF	induce apoptosis, prevent celldifferentitation	[11, 31]
Endothelial cells	prevent NF-κB translocation	DMF	TNF α↓, ICAM-1↑, E-selectin↑ VCAM-1↑	[4, 27]
Glia cells	TNF-α↓, IL-1β↓, IL-6↓	DMF	NQO-1↑,cellular Glutathion↑, NO↓	[30]

**Table 2:** Clinical and MRI Data of a Phase II Study with BG-12 in RRMS

End Point MRI	Placebo	BG12		
		120 mg qd	120 mg tid	240 mg tid
Mean number of new Gd+ lesions (Weeks 12-24)	4.5	3.3	3.1	1.4*
Mean number of new or enlarging T2-hyperintense lesions, % of patients (Week 24)	4.2	3.8	4.1	2.2*
Mean number of new T1-hypointense lesions, % of patients (Week 24)	1.7	1.3	1.5	0.8*
Clinical Data (weeks 0-24)				
Annualized relapse rate	0.65	0.42	0.78	0.44
Relapse free, % of patients	69	78	64	71

* significant result.
